# Projection of mesothelioma mortality in Britain using Bayesian methods

**DOI:** 10.1038/sj.bjc.6605781

**Published:** 2010-07-13

**Authors:** E Tan, N Warren, A J Darnton, J T Hodgson

**Affiliations:** 1 Health and Safety Laboratory, Harpur Hill, Buxton, Derbyshire SK17 9JN, UK; 2 Epidemiology Unit, Health and Safety Executive, Redgrave Court, Merton Road, Bootle, Merseyside L20 7HS, UK

**Keywords:** mesothelioma, mortality, Great Britain, Bayesian, Markov Chain Monte Carlo

## Abstract

**Background:**

Mesothelioma mortality has increased more than ten-fold over the past 40 years in Great Britain, with >1700 male deaths recorded in the British mesothelioma register in 2006. Annual mesothelioma deaths now account for >1% of all cancer deaths. A Poisson regression model based on a previous work by Hodgson *et al* has been fitted, which has allowed informed statistical inferences about model parameters and predictions of future mesothelioma mortality to be made.

**Methods:**

In the Poisson regression model, the mesothelioma risk of an individual depends on the average collective asbestos dose for the individual in a given year and an age-specific exposure potential. The model has been fitted to the data within a Bayesian framework using the Metropolis–Hastings algorithm, a Markov Chain Monte Carlo technique, providing credible intervals for model parameters as well as prediction intervals for the number of future cases of mortality.

**Results:**

Males were most likely to have been exposed to asbestos between the ages of 30 and 49 years, with the peak year of asbestos exposure estimated to be 1963. The estimated number of background cases was 1.08 cases per million population.

**Conclusion:**

Mortality among males is predicted to peak at approximately 2040 deaths in the year 2016, with a rapid decline thereafter. Approximately 91 000 deaths are predicted to occur from 1968 to 2050 with around 61 000 of these occurring from 2007 onwards.

Mesothelioma is a cancer that mainly affects the pleura (the membrane that covers the lungs and lines the internal chest wall) and the peritoneum (the membrane that forms the lining of the abdominal cavity). Of all deaths, 85% have been among men and the majority of these were caused by occupational exposure to asbestos fibres (Rake *et al*, 2009). The disease has a long latency period; symptoms usually emerge between 15 and 60 years after exposure to asbestos, after which mesothelioma is rapidly fatal. The majority of deaths occur among those >60 years of age, with few deaths occurring among those <50 years. Although the majority of cases of mesothelioma are caused by exposure to asbestos, much of which occurred in occupational settings, particularly among men, a small number of cases occur spontaneously among those with no history of exposure. Annual mesothelioma deaths have increased more than ten-fold over the past 40 years and now account for >1% of all cancer deaths.

Predictions of mesothelioma mortality have been made in several countries. Clements *et al* (2007) modelled mesothelioma mortality in Australia using both an age–birth cohort model and a model based on that introduced by Hodgson *et al* (2005). Banaei *et al* (2000) predicted mortality in France using a method based on a risk function that links mortality with past exposure to asbestos. Segura *et al* (2003) used an age–period–cohort model to predict mortality in the Netherlands. Hodgson *et al* (2005) developed a model based on the estimated collective population exposure to asbestos and a specific form for the relationship between mesothelioma risk and time since first exposure at the population level. The model was fitted to mesothelioma mortality for Great Britain up to 2001 and projections of mortality were made by applying fitted mesothelioma rates to future population projections. Confidence intervals for the parameters and prediction intervals for future estimated annual deaths could not be made because of limitations in the optimisation approach adopted. This paper presents the results of a Bayesian statistical analysis to refit a modified version of this model using updated mortality data up to 2006 and a Markov Chain Monte Carlo (MCMC) method of computation. The Bayesian approach allowed credible and prediction intervals to be calculated; thus, informed statistical inferences about model parameters and predictions of future mortality, in particular the scale and timing of the peak in deaths, could be made.

## Materials and methods

### The model

The British Mesothelioma Register contains all deaths in Great Britain since 1968 in which mesothelioma was mentioned on the death certificate. It provides the basis for a consistent data series of mesothelioma mortality over nearly four decades. In both males and females, 99% of all these deaths are among those between the ages of 20 and 89 years. The data used in the analyses carried out in this report are based on deaths of males aged 20–89 years, between 1968 and 2006. The model developed by Hodgson *et al* (2005) is based on that developed by the Health Effects Institute (1991). In brief, the Health Effects Institute (1991) model, in which an individual's mesothelioma risk is assumed to be proportional to the increase in cumulative exposure multiplied by a power of time since exposure lagged by 10 years, was applied at the population level; this was achieved by assuming that the average asbestos exposure for males in Great Britain in each year can be summarised by a single estimate and that their exposure in any given year also depends on their age. Parameters to model change in completeness of mesothelioma diagnosis over time and the clearance half-life of asbestos fibres in the lungs were also included. Allowance for a background rate of mesotheliomas not caused by asbestos exposure was not originally included by Hodgson *et al* (2005); however, background cases may account for a higher proportion of deaths in certain years and among the most recent birth cohorts, and we have included a term to allow for such cases in the modified version of the model that can be represented as follows: 
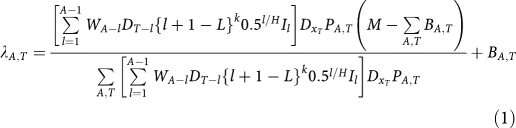
 where *λ*_*A*,*T*_ is the number of deaths at age *A* in year *T*, *W*_*A*_ is the overall age-specific exposure potential at age *A*, *D*_*T*_ is the overall population exposure in year *T*, 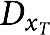 is the proportion of mesothelioma deaths in year *T* that are recorded, *L* is the lag period in years between exposure and disease occurrence, *H* is the half-life in years for asbestos clearance from the lungs, *k* is the power of time representing the increase of risk with increase of time since exposure, *P*_*A,T*_ is the person-years at risk for age *A* in year *T*, *M* is the total observed mesothelioma deaths from 1968 to 2006, *I* is an indicator variable where *I*=0 if *l*<*L–*1 and *I*=1 otherwise, *l* indexes years lagged from the risk year and *B*_*A,T*_ is the number of background cases for age *A* at year *T*.

### Modelling technique

The model was originally fitted by Hodgson *et al* (2005) using a manual iterative approach to minimising the model deviance. In this case, the deviance can be expressed as 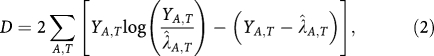 where *Y*_*A,T*_ are the observations and *λ*_*A,T*_ are the fitted values. However, confidence intervals for both the parameter estimates and predictions of mortality could only be obtained using an informal numerical approach rather than analytically. We used a more refined statistical analysis using the Metropolis–Hastings algorithm (Metropolis *et al*, 1953; Hastings, 1970), an MCMC method. This method allows not only model parameters to be estimated, but also Bayesian credibility intervals to be easily obtained using formal statistical methods, which is the main advantage of adopting a Bayesian approach. Because of the number of parameters and the complexity of the model, it would have been difficult to adopt a frequentist approach to obtain parameter estimates and confidence intervals.

### Model parameters

The age-specific exposure potential, *W*_*A*_, allowed the exposure of males in a given year to vary by age. Nine parameters were assigned to *W*_*A*_, representing the exposure weighting for the age groups 0–4, 5–15, 16–19, 20–29, 30–39, 40–49, 50–59, 60–64 and 65+, with the age group 20–29 years chosen as the baseline category.
The overall population exposure, *D*_*T*_, represents the average ‘effective carcinogenic dose’ in the breathing zone of men aged 20–89 years and is included as a unit-free parameter vector in the model. The shape of the exposure curve and the change in exposure levels over time is the main interest in the inclusion of *D*_*T*_. *D*_*T*_ was defined by growth and decline rates for years in multiples of 10 before and after the maximum exposure year, called *Peakyear* (at which the gradient of the exposure curve is zero). The growth rates for intermediate years were determined by linear interpolation. The set of growth rates at *Peakyear* −65, *Peakyear* −55, *Peakyear* −45, *Peakyear* −35, *Peakyear* −25, *Peakyear* −15, *Peakyear* −5, *Peakyear* +5 and *Peakyear* +15 was included as a multivariate parameter in the model. Parameters defining the exposure distribution in the most recent years could not be estimated because of the long latency period of mesothelioma; instead, assumptions about the level of exposure from the year 2000 based on the HSE Regulatory Impact Assessment (HSE, 2002) were used as in Hodgson *et al* (2005). From the year 2000 onwards, it was assumed that the total asbestos exposure to the population would be approximately 4% of the peak value in 2000, 2% in 2010 and 0.75% in 2050. Between the last year for which the growth rate was estimated and 2000, the value of the exposure was determined by linear interpolation.
The diagnostic trend 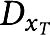 was defined by a parameter *α*, representing the annual percentage decrease in the number of missed cases, working backwards in time from 1997, in which diagnosis was assumed to be essentially complete (98%).
The background rate is represented by the number of cases per million in the male population. The age distribution of the background cases in each year is assumed to be (*A*−*L*)^*k*^. The proportion of background cases at age *A* in each year is therefore assumed to be 
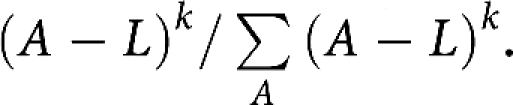
.

### Population projections

To obtain mesothelioma mortality projections from 2007 to 2050, population projections of the number of males aged between 20 and 89 at every year between 2007 and 2050 were obtained from the Office for National Statistics.

### The model

From a Bayesian perspective, the parameters of a statistical model are considered random quantities. Bayesian inference can usually be summarised by random draws from the posterior distributions of the model parameters. Let Lik*(Y∣θ)* be the likelihood function of the data *Y*, *θ* be the vector of model parameters and *ϕ(θ)* be the prior distribution of the parameters, which represents the prior information we have on *θ*. The posterior distribution *π(θ)* of *θ* is 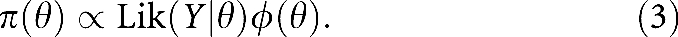


Assuming that the observations follow a Poisson distribution, the likelihood function is 
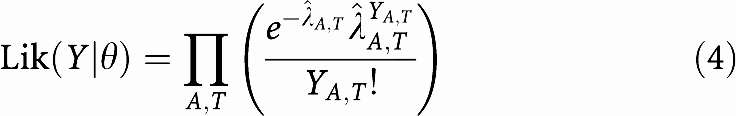
 which is the product of the individual likelihood contributions for each observation over all ages and years of death. We assumed that all possible parameter values within a chosen interval were equally likely by using uniform prior distributions. The intervals were chosen by considering the maximum plausible ranges, taking into account the results of Hodgson *et al* (2005).

### Statistical inference

Unfortunately, because of the complexity of the likelihood, the posterior distribution is unavailable in closed form. Numerical techniques, particularly MCMC, are thus required to evaluate the posterior distribution. Thus, MCMC techniques require simulation to generate random samples from a complex posterior distribution. We generated 35 000 sets of random draws from the joint posterior distribution of the model parameters after a burn-in of 20 000 iterations to minimise the effect of initial values on posterior inference. After the burn-in period, the empirical distributions should eventually approximate the true shapes of the posterior distributions closely. Point estimates and credible intervals are then calculated from this distribution.

### Proposal distributions

Apart from the proposal distribution for *Peakyear*, each distribution was chosen to be normal with s.d. such that the acceptance probability ranged between 20 and 45%. It should be noted that the proposal distributions do not have an effect on the posterior parameter estimates, but only on the convergence, mixing and autocorrelation of the chains generated by the Metropolis–Hastings algorithm. The Metropolis–Hastings algorithm was implemented in Matlab (The MathsWorks, Inc., Natick, MA, USA; 2008).

### Prior distributions

Non-informative prior distributions for each parameter were chosen by considering plausible ranges, taking into account the results in Hodgson *et al* (2005). In the previous analysis, *k* has been estimated at between 2 and 3. It was unlikely that the risk decreased with time since exposure, and hence the prior for *k* was chosen to be *U*(0, 10). Each of the *W* parameters represents age-specific exposure potential and can only take positive values. It was considered unlikely that the risk in any of the age groups was 10 times greater than that of males aged 20–29 years (the baseline age group), and hence the priors for *W* were chosen to be *U*(0, 10). Each of the *D* parameters represents the growth rates of population exposure levels. As the overall population exposure can only take positive values the decline rate must not exceed 100%, and hence the lower bound for *D* must be −100. Taking into account the data on asbestos imports as well as the levels of asbestos use in Great Britain, the peak year of exposure was assumed to be between 1950 and 2000, and hence the prior distribution of *Peakyear* was chosen to be uniformly distributed on integer values between 1950 and 2000. By definition, the background rate can only take positive values. Hodgson *et al* (2005) suggest that a background rate of 1 to 2% of total mesothelioma deaths, equating to approximately 25–50 male deaths annually, is widely assumed. A uniform *U*(0, 20) prior was chosen for *α* (cases per million). As a result of problems encountered when attempting to estimate *H*, various prior distributions for *H* were considered. Table 1 shows the prior distributions that were used.

## Results

The parameter estimates are shown in Table 2. The parameter *L* was fixed at 10, as in Hodgson *et al* (2005).

### Exponent of time

The posterior median of the exponent of time, *k*, was 2.42.

### Half-life

Increasing the clearance half-life parameter *H* was found to improve model fit in preliminary analysis; however, convergence in the posterior distribution for *H* was unattained after several thousand iterations of the MCMC algorithm, suggesting that there is no finite optimal value of *H*. In light of this, *H* was fixed at 1 000 000 years in the final model, corresponding to virtually no clearance of asbestos from the lungs. However, there was high negative correlation between *H* and *k*; fixing *k* to larger values led to lower simulated samples of *H*. It was thus difficult to obtain a value of *H* that is close to the true value. The large half-life value of 1 000 000 years was selected as it resulted in a better fit than a lower value; however, it should be interpreted with caution.

### Diagnostic trend

The inclusion of the diagnostic trend component in the model did not seem to improve the fit of the model. Although the best fitting model was one in which the diagnostic trend component was excluded, this does not necessarily imply that the proportion of missed cases remains unchanged over time; changes may have been encapsulated in the population exposure profile or the background case component of the model.

### Age-specific exposure potential

The estimates of the age-specific exposure potential parameters suggested that this was highest among males aged 30 to 49 years, with males aged <15 years and >50 years least likely to be exposed. Convergence of the exposure time profile parameters could not be achieved when all of these were included as estimated parameters.

### Exposure profile

Along with the peak exposure year (1963), sharp local peaks in the exposure profile were also present at *Peakyear* −35 and *Peakyear* −15. Attempts were made to smooth the exposure profile before the peak year by changing the assumptions of the population exposure before *Peakyear* −45. However, several attempts resulted in the rate of change in exposure levels at *Peakyear* −45 increasing and failing to converge, as well as the rate at *Peakyear* −35 eventually taking up negative values. In light of this, the rates of change in exposure levels at *Peakyear* −65, *Peakyear* −55 and *Peakyear* −45 were fixed at 0, 1000 and 100 0000, respectively. The estimated exposure curve in the final model indicated a high level of exposure around *Peakyear* −35, soon followed by a sharp decrease in exposure. A rapid increase in population exposure followed from the 1940s to the mid-1960s, reaching a maximum in 1963 and decreasing thereafter.

### Background rate

The background rate was estimated at 1.08 cases per million, corresponding to approximately 23 cases in 2006 among males aged 20 to 89 years.

**Figure 1:**
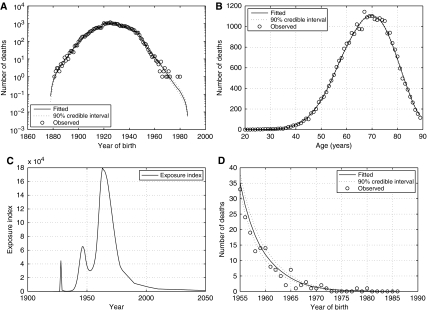
Metropolis–Hastings algorithm. (**A**) Observed and fitted deaths by year of birth. (**B**) Observed and fitted deaths by age. (**C**) Derived exposure index. (**D**) Observed and fitted deaths for 1955–1985 birth cohorts.

Figure 1A–D shows plots of observed and fitted deaths by year of birth, age of death and year of death for males aged between 20 and 89 years.

### Projections

**Figure 2:**
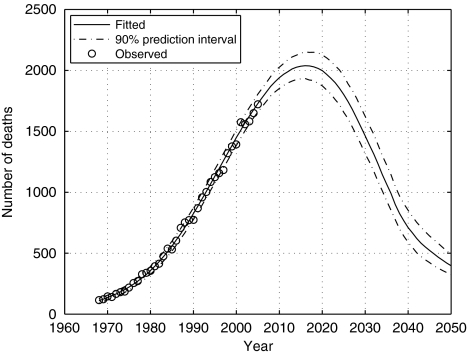
Observed deaths with fitted 50th percentile curve and 90% prediction interval.

The peak year of mesothelioma mortality among males aged between 20 and 89 years was projected to be 2016 (90% CI: 2015–2017). Although only very few deaths have occurred in males <20 years or >89 years, this estimate has been rescaled to give the total number of deaths among males of all ages. Predicted ratios, projections and credible intervals for mesothelioma mortality in all males are given in Table 3. The estimate of the peak number of deaths in all males is 2038 (90% prediction interval 1929–2156) in the year 2016 (90% CI: 2015–2016). A rapid decline in cases is expected after the peak year. Approximately 91 000 deaths are predicted to occur from 1968 to 2050 with around 61 000 of these occurring from 2007 onwards. Figure 2 shows a plot of fitted and observed deaths by year of death, along with a 90% prediction interval for all male deaths.

### Lag period

It has been shown that for many animal tumours, cancer incidence rises approximately as some power of age or time since first exposure to a carcinogen (Doll, 1971). Peto *et al* (1995) fitted a model of this type to mesothelioma mortality data for North American insulation workers and showed that incorporating a lag of 10 years from asbestos exposure to disease occurrence provided a better fit than assuming no lag period (Peto and Selikoff, 1982). A lag of 10 years has subsequently been assumed by several researchers (Hodgson *et al*, 2005) and has also been assumed in this paper. Although there is a significant body of work that supports a lag time of 10 years, a fixed lag does represent a strong assumption. Therefore, a local sensitivity analysis of the lag time was carried out to test the sensitivity of model results to this assumption. Lag times between 0 and 15 years were considered. Results showed that both the peak year and the peak number of deaths were sensitive to the assumed lag. In particular, a lag of 0 resulted in a peak year further into the future, whereas a lag of 15 years moved the peak closer to present time. However, the deviances calculated under these lags indicated a much poorer fit to the data than under a lag of 10 years. The peak year and peak number of deaths were not sensitive to small variations in about a lag of 10 years.

### Model adequacy

The deviance residuals may be used to measure the fit of the Poisson model and are defined as 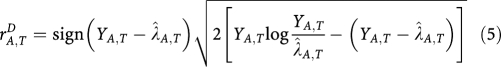 where *r*^*D*^_*A,T*_ is the contribution to the deviance of the observation at age *A* and year *T* and 
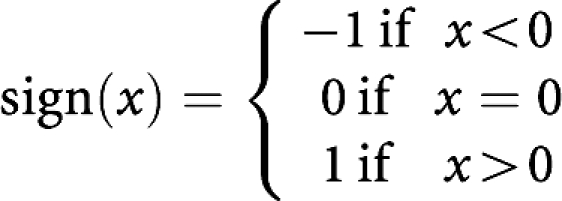


The distribution of the deviance residuals is approximately normal. For a good fit, approximately 95% of the deviance residuals should lie in the range [−2, 2]. Out of the 126 deviance residuals resulting from fitting the model, 121 (96%) lie in the range [−2, 2] when using the posterior medians of the Metropolis–Hastings algorithm, suggesting a satisfactory fit.

## Discussion

In this analysis we found that a modified version of the model developed by Hodgson *et al* (2005) provided a good statistical fit to mesothelioma data from 1968 to 2006, suggesting that it provides a sound basis for shorter-term projections of future levels of mortality. Updated projections are broadly in agreement with those based on earlier analyses.

The use of an improved optimisation approach based on MCMC and Bayesian techniques provided increased confidence than in previous analyses that the best fitting model was obtained, as well as allowed a more thorough assessment of model adequacy and model parameters to be made. It has also allowed the construction of prediction intervals for the future mortality to be made.

Fitting the model revealed that the profile of estimated population asbestos exposure over time had several local maxima, whereas in previous analyses the profile increased monotonically before the peak year and decreased monotonically thereafter. The estimate of the global peak year of exposure was 1963 with local peaks around 1930 and 1950, after which exposure rapidly decreased. These peaks coincided with specific events that took place in Great Britain that were likely to have had an effect on the use of asbestos, which may explain the observed pattern to some extent, although it seems unlikely that actual changes in population exposure would have been as extreme. The first peak coincides with the Great Depression as well as the establishment of Asbestos Industry Regulations in 1931, which were introduced in Great Britain to regulate the use of asbestos in the asbestos industry, although this sharp peak may have been exaggerated by the assumptions on exposure growth rates in earlier years. Even when these assumptions were relaxed, the pattern of convergence in the parameter estimates representing the exposure growth rates in earlier years still indicated a peak around 1930. This peak, however, only contributes to a small proportion of mesothelioma deaths. When World War II took place between 1939 and 1945, shipbuilding in naval yards increased as the industry saw a large increase in demand. Use of asbestos in naval yards was likely to have increased during that period. After the war, when demand fell, shipbuilding activity decreased and thus the use of asbestos in naval yards was likely to have decreased. The second local peak of asbestos exposure coincides with this post-war reduction in shipyard activity. These features of the population exposure curve persist when refitting the model to observations of mortality to 2001 (the basis for earlier analyses). This suggests that differences between the updated exposure profile and that of Hodgson *et al* (2005) are because of the improved model-fitting approach rather than because of refinements to the model and additional observations of mortality. As mesothelioma is usually only diagnosed several decades after exposure to asbestos, and as the peak year of mortality has yet to be reached, there is greater uncertainty in the estimated exposure profile from mid-1960s onwards.

In our model, the last year for which the population exposure was estimated was 1978. The extent of the population exposure beyond this point has limited effect on the predicted mesothelioma deaths within the range of years for which observations of mortality are available (up to 2006), and thus on the model fit. Furthermore, predictions of the scale and timing of the peak number of mesothelioma deaths are not highly dependent on exposure after the late 1970s. However, the shape of the exposure curve after 1978 is required to use the model to make longer-term predictions. Some limited investigation of different exposure curves suggested that a levelling off of the exposure in the late 1970s provides a marginally better fit than a continuing very steep decline in exposure. However, such considerations cannot be used as grounds for preferring one exposure curve over another. Decisions regarding the shape of the exposure profile in this region must draw on other sources of evidence about the extent of population exposure more recently.

For our projections we used the same assumptions about exposure beyond the year 2000 as in Hodgson *et al* (2005), and additionally assumed a linear decline in exposure between 1978 and 2000. However, the prediction intervals of our projections incorporate only the uncertainty in the fitted model parameters, and not the unquantifiable but potentially considerable degree of additional uncertainty arising from the particular chosen shape of the exposure curve beyond 1978. For example, if the population exposure levelled off in 1978 and then continued indefinitely at this level rather than continuing to decline, as we have assumed, the model predicts a much slower decline in mortality after the peak year, and consequently much larger estimates of the total mortality to year 2050 that exceed those based on our upper prediction interval.

Although this analysis confirmed that the current model provides a good fit to the observations of mesothelioma mortality to date, and provides a reasonable basis for projections in the short term, it is much less clear whether it provides a good basis for longer-term projections, even if we could be more confident about the exposure curve beyond 1978. Male mortality to date is still dominated by the effect of substantial past occupational exposures, and in these circumstances this model, in which mesothelioma risk depends on a power of time since first exposure, seems to fit the data well.

The estimated number of background cases was 1.08 cases per million population, equivalent to 23 cases in 2006 among males aged 20–89 years. This is in good agreement with the value of 1 to 2% of total cases as suggested by McDonald and McDonald (1996), who carried out backward extrapolation of mesothelioma mortality trends from epidemiological studies from various countries; a figure of 1.15% (95% confidence interval 0.90–1.45) has been suggested by Teta *et al* (2008) for males, based on US mesothelioma patterns between 1973 and 2002. Although the proportion of background cases in recent years among males has been small compared with the relatively large number of asbestos-related cases, the background cases will represent a larger proportion of all cases in future years when the number of asbestos-related cases will decline.

Hodgson *et al* (2005) included a diagnostic trend parameter in their models, which was estimated at 5% in their non-clearance model. The results of our analyses suggested little statistical evidence for a diagnostic trend and no apparent improvement to the fit of the model. Although the best fitting model was one in which the diagnostic trend component was excluded, this does not necessarily imply that the proportion of missed cases has remain unchanged over time; changes in the proportion of missed cases over time may have been encapsulated in the population exposure profile or the background case component of the model.

The peak number of mesothelioma deaths among males aged 20–89 years reported in Hodgson *et al* (2005) was approximately 1846 deaths between 2011 and 2015 based on data up to 2001, which is lower than the peak of 1990 (90% prediction interval 1886–2100) deaths among males ages 20–89 years in the year 2016 predicted in this study. However, more than half of the difference in the peak number of deaths is because of the use of updated projections of the future British population.

Different estimates of peak mesothelioma mortality have been predicted in other countries; in Australia, the peak is expected at approximately 700 cases per year in 2010 (Leigh and Driscoll, 2003). In France, the peak of approximately 2200 cases per year is expected some time after 2020 (Ilg *et al*, 1998), whereas in the Netherlands (Segura *et al*, 2003), up to 900 cases per year of pleural mesothelioma are expected around the year 2028. These projections, among others that have been made for mesothelioma mortality in Europe, indicate that although the number of deaths has been rapidly increasing in recent years, mortality may not reach a peak for several years. Although the pattern of projected future mesothelioma mortality in Britain is broadly consistent with these predictions for other industrialised countries, the scale of mesothelioma mortality varies between countries and rates in Britain are among the highest worldwide (Rake *et al*, 2009). This is a reflection of large-scale importation and use of asbestos particularly in building products. The assumptions that we have made about future exposures are uncertain and the contribution they will make to the cumulative total burden of mesothelioma in the long run will ultimately depend on the effectiveness of current controls to prevent inadvertent exposures because of maintenance activities in buildings that still contain asbestos products.

## Figures and Tables

**Table 1:** Prior distributions for model parameters

**Parameter**	**Prior**
*H*	Various
*k*	*U*(0, 10)
*W*_*k*_ *∀k*	*U*(0, 10)
*D*_*k*_ *∀k*	*U*(−100, 200)
*α*	*U*(−0.07, 0.09)
*Peakyear*	*U*(1950, 2000)
*Rate*	*U*(0, 20)

**Table 2:** Parameter estimates from fitting Poisson regression model

**Parameter**	**Metropolis–Hastings** **Posterior median (90% CI)**	**Hodgson *et al* (2005) Estimate**
Power of time since exposure, *k*	2.42 (2.28, 2.56)	2.6
Background rate, *Rate* (per million)	1.08 (0.71, 1.51)	—
Maximum exposure year, *Peakyear*	1963	1967
Clearance half-life, *H* (years)	1 000 000 (fixed)	1000 (fixed)
Diagnostic trend, *α* (%)	—	5
		
*Change in exposure index (percentage per year) in peak year±X years*		
−65	0 (fixed)	—
−55	1000 (fixed)	—
−45	100 000 (fixed)	29
−35	−91.3 (−98.2, −50.1)	6
−25	104.6 (44.8, 135.5)	11
−15	−25.5 (−34.9, −8.28)	9
−5	36.6 (23.2, 47.8)	5
0 (Peak year)	0 (by definition)	0 (by definition)
5	−7.5 (−14.1, −1.4)	−14
15	−18.6 (−27.5, −8.8)	−39
		
*Relative exposure potential for age group (years)*		
0–4	0.0019 (0.0001, 0.0074)	0.00
5–15	0.0023 (0.0002, 0.0091)	0.03
16–19	0.25 (0.048, 0.393)	0.21
20–29	1.00 (baseline)	1.00
30–39	1.79 (1.51, 2.03)	1.24
40–49	1.59 (1.25, 1.94)	1.11
50–59	0.13 (0.01, 0.41)	0.00
60–64	0.56 (0.06, 1.54)	0.00
65+	0.42 (0.03, 1.56)	0.00

**Table 3:** Projections of all male mesothelioma deaths

**Year**	**Male 20–89 years** **Projection (90% prediction interval)**
2009	1910 (1827, 1993)
2010	1941 (1855, 2026)
2011	1968 (1870, 2059)
2012	1993 (1897, 2084)
2013	2012 (1913, 2106)
2014	2027 (1926, 2129)
2015	2035 (1929, 2141)
2016	2038 (1928, 2156)
2017	2037 (1928, 2147)
2018	2031 (1912, 2152)
2019	2017 (1903, 2141)
2020	1997 (1871, 2132)
2030	1462 (1314, 1626)
2040	708 (588, 851)
2050	396 (326, 487)

## References

[bib1] Banaei A, Auvert B, Goldberg M, Gueguen A, Luce D, Goldberg S (2000) Future trends in mortality of French men from mesothelioma. Occup Environ Med 57: 488–494 10854503 10.1136/oem.57.7.488 PMC1739977

[bib2] Clements M, Berry G, Shi J, Ware S, Yates D, Johnson A (2007) Projected mesothelioma incidence in men in New South Wales. Occup Environ Med 64: 747–752 17449562 10.1136/oem.2006.031823 PMC2078410

[bib3] Doll R (1971) The age distribution of cancer: implications for models of carcinogenesis. J R Stat Soc Ser A 134: 133–166

[bib4] Hastings WK (1970) Monte Carlo sampling methods using Markov chains and their applications. Biometrika 57: 97–109

[bib5] Health and Safety Executive (2003) Mesothelioma Mortality in Great Britain: Estimating the Future Burden. Health and Safety Executive: Bootle, UK

[bib6] Health Effects Institute (1991) Asbestos in Public and Commercial Buildings: A Literature Review and Synthesis of Current Knowledge. Health Effects Institute – Asbestos Research: Cambridge, MA

[bib7] Hodgson JT, McElvenny DM, Darnton AJ, Price MJ, Peto J (2005) The expected burden of mesothelioma mortality in Great Britain from 2002 to 2050. Br J Cancer 92: 587–593 15668716 10.1038/sj.bjc.6602307 PMC2362088

[bib8] HSE (2002) Amendment to the Control of Asbestos at Work Regulations 1987 and AcoP: Regulatory Impact Assessment. Health and Safety Executive: Bootle, UK

[bib9] Ilg AG, Bignon J, Valleron AJ (1998) Estimation of the past and future burden of mortality from mesothelioma in France. Occup Environ Med 55: 760–765 9924453 10.1136/oem.55.11.760 PMC1757524

[bib10] Leigh J, Driscoll T (2003) Malignant mesothelioma in Australia, 1945–2002. Int J Occup Environ Health 9: 206–217 12967156 10.1179/oeh.2003.9.3.206

[bib11] McDonald JC, McDonald AD (1996) The epidemiology of mesothelioma in historical context. Euro Respir J 9: 1932–1942 10.1183/09031936.96.09091932 8880114

[bib12] Metropolis N, Rosenbluth AW, Rosenbluth MN, Teller AH, Teller E (1953) Equations of state calculations by fast computing machines. J Chem Phys 21: 1087–1092

[bib13] Peto J, Hodgson JT, Matthews FE, Jones JR (1995) Continuing increase in mesothelioma mortality in Britain. Lancet 345: 535–539 7776771 10.1016/s0140-6736(95)90462-x

[bib14] Peto J, Selikoff IJ (1982) Mesothelioma mortality in asbestos workers: implications for models of carcinogenesis and risk assessment. Br J Cancer 45: 124–135 7059455 10.1038/bjc.1982.15 PMC2010947

[bib15] Rake C, Gilham C, Hatch J, Darnton A, Hodgson J, Peto J (2009) Occupational, domestic and environmental mesothelioma risks in the British population: a case-control study. Br J Cancer 100: 1175–1183 19259084 10.1038/sj.bjc.6604879 PMC2669989

[bib16] Segura O, Burdorf A, Looman C (2003) Update of predictions of mortality from pleural mesothelioma in the Netherlands. Occup Environ Med 60: 50–55 12499457 10.1136/oem.60.1.50 PMC1740374

[bib17] Teta MJ, Mink PJ, Lau E, Sceurman BK, Foster ED (2008) US mesothelioma patterns 1973-2002: indicators of change and insights into background rates. Eur J Canc Prev 17: 525–534 10.1097/CEJ.0b013e3282f0c0a2 18941374

